# Expression of concern: Evaluating the biological characteristics of targeted ZIF-8-encapsulated individual and combined drug systems for enhanced *in vivo* toxicity mitigation using folic acid ligands

**DOI:** 10.1039/d6ra90041k

**Published:** 2026-04-10

**Authors:** Noor S. Sadeq, Mas Jaffri Masarudin, Mohd Basyaruddin Abdul Rahman, Suet Lin Chia, Syahida Ahmad, Haslina Ahmad

**Affiliations:** a Department of Chemistry, Faculty of Science, Universiti Putra Malaysia, UPM 43400 Serdang Selangor Malaysia haslina_ahmad@upm.edu.my noor.s.sadeq@uotechnology.edu.iq; b Medical & Industrial Materials Branch, Applied Science Department, University of Technology Baghdad Iraq; c Department of Cell and Molecular Biology, Faculty of Biotechnology and Biomolecular Sciences, Universiti Putra Malaysia Serdang 43400 Selangor Malaysia; d Nanomaterials Synthesis and Characterisation Laboratory, Institute of Nanoscience and Nanotechnology, Universiti Putra Malaysia Selangor Malaysia; e Integrated Chemical BioPhysics Research, Faculty of Science, Universiti Putra Malaysia (UPM) Serdang 43400 Selangor Malaysia; f UPM-MAKNA Cancer Laboratory, Institute of Bioscience, Universiti Putra Malaysia Serdang 43400 Selangor Malaysia; g Department of Microbiology, Faculty of Biotechnology and Biomolecular Science, Universiti Putra Malaysia, UPM 43400 Serdang Selangor Malaysia; h Department of Biochemistry, Faculty of Biotechnology and Biomolecular Science, Universiti Putra Malaysia, UPM 43400 Serdang Selangor Malaysia

## Abstract

Expression of concern for ‘Evaluating the biological characteristics of targeted ZIF-8-encapsulated individual and combined drug systems for enhanced *in vivo* toxicity mitigation using folic acid ligands’ by Noor S. Sadeq *et al.*, *RSC Adv.*, 2026, **16**, 1912–1931, https://doi.org/10.1039/D5RA07756G.

The Royal Society of Chemistry is publishing this expression of concern in order to alert readers that concerns have been raised regarding the interpretation of the FTIR data in Fig. 1 and the reliability of the survival rate data in Fig. 8.

An update will be provided as soon as possible.

Laura Fisher

2nd April 2026

Executive Editor, *RSC Advances*

